# 324. COVID-19-Associated Pulmonary Aspergillosis (CAPA) at Veterans Affairs (VA) Hospitals in Southern California and Arizona

**DOI:** 10.1093/ofid/ofab466.526

**Published:** 2021-12-04

**Authors:** Michelle Fang, Phuong Khanh Nguyen, Tony T Chau, Ashley Doan, Andrew S Varker, Marshall T Renna, Raul Nakamatsu, Christopher J Graber, Matthew B Goetz, Matthew B Goetz, Martin Hoenigl, Martin Hoenigl, Sanjay Mehta, Scott T Johns

**Affiliations:** 1 VA San Diego Healthcare System, Temple City, California; 2 VA Greater Los Angeles Healthcare System, Los Angeles, California; 3 Veteran Affairs Healthcare Systems, Loma Linda, CA; 4 VA Loma Linda Healthcare System, Loma Linda, California; 5 Phoenix VA Health Care System, GIlbert, AZ; 6 Southern Arizona VA Health Care System, Tucson, Arizona; 7 Loma Linda VA Healthcare System, Loma Linda, California; 8 VA Greater Los Angeles Healthcare System/UCLA, Los Angeles, California; 9 VA Greater Los Angeles Healthcare System and David Geffen School of Medicine at UCLA, VA-CDC Practice-Based Research Network, Los Angeles, California; 10 UC San Diego, San Diego, California; 11 University of California San Diego, San Diego, California; 12 San Diego VA Healthcare System, San Diego, California

## Abstract

**Background:**

The data on CAPA in the U.S. are limited to date and clinical characteristics unique to this phenomenon have not been widely reported.

**Methods:**

This retrospective observational study was conducted at multiple VA hospitals across southern California and Arizona. CAPA cases were identified in inpatients with laboratory-confirmed COVID-19 based on microbiologic or serologic evidence of aspergillosis and pulmonary abnormalities on imaging, and were classified according to ECMM/ISHAM consensus definitions. Characteristics of interest included immunosuppressive/modulatory agents used prior to onset of CAPA, COVID-19 disease course, length of hospitalization, and mortality.

**Results:**

Seventeen patients with probable (18%) or possible (82%) CAPA were identified from April 2020 to March 2021. Values below reported as medians. All patients were male and 13 (76%) were white, with age 74 years and BMI 26 kg/m^2^. Baseline comorbidities included diabetes mellitus (47%), cardiovascular disease (65%), and pulmonary disease (71%). Evidence of aspergillosis was mostly based on respiratory culture, with mainly *A. fumigatus* (75%). Systemic corticosteroids were used in 14 patients, with a total dose of 400 mg prednisone equivalents starting 10 days prior to *Aspergillus* detection. Patients also received tocilizumab (18%), leflunomide (6%), tacrolimus (6%), mycophenolate (6%), and investigational agent LSALT or placebo (6%); 2 patients (12%) did not receive any immunosuppression/modulation. Length of hospitalization for COVID-19 was 22 days. Death occurred in 12 patients (71%), including all patients with probable CAPA, at 34 days after COVID-19 diagnosis and 16 days after CAPA diagnosis. Eight patients (47%) were treated for aspergillosis; mortality did not appear to differ with treatment (75% vs. 67%).

Table 1. COVID-19 Inpatient Characteristics

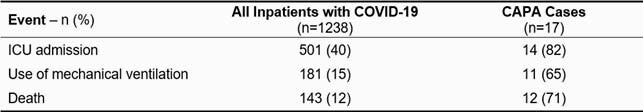

Table 2. Incidence of Aspergillus Growth on Respiratory Culture

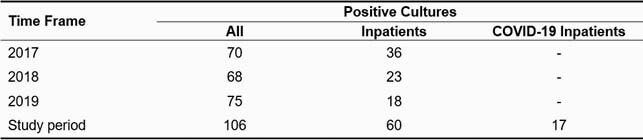

**Conclusion:**

This case series reports high mortality among patients with CAPA; the primary contributor to this outcome is unclear. Frequency of lower respiratory tract sampling in patients with COVID-19 may have limited diagnosis of CAPA. Interestingly, inpatient respiratory cultures with *Aspergillus* spp. increased compared to previous years. Future work will attempt to identify risk factors for CAPA and attributable mortality via comparison to inpatients with COVID-19 without CAPA.

**Disclosures:**

**Matthew B. Goetz, MD**, Nothing to disclose **Martin Hoenigl, MD**, **Astellas** (Grant/Research Support)**Gilead** (Grant/Research Support)**Pfizer** (Grant/Research Support) **Martin Hoenigl, MD**, Astellas (Individual(s) Involved: Self): Grant/Research Support; F2G (Individual(s) Involved: Self): Grant/Research Support; Gilead (Individual(s) Involved: Self): Grant/Research Support; Pfiyer (Individual(s) Involved: Self): Grant/Research Support; Scýnexis (Individual(s) Involved: Self): Grant/Research Support **Sanjay Mehta, MD, D(ABMM), DTM&H**, **MedialEarlySign** (Consultant)**ZibdyHealth** (Employee, Medical Officer - Unpaid)

